# Transforming Pt-SnO_2_ Nanoparticles into Pt-SnO_2_ Composite Nanoceramics for Room-Temperature Hydrogen-Sensing Applications

**DOI:** 10.3390/ma14092123

**Published:** 2021-04-22

**Authors:** Ming Liu, Caochuang Wang, Pengcheng Li, Liang Cheng, Yongming Hu, Yao Xiong, Shishang Guo, Haoshuang Gu, Wanping Chen

**Affiliations:** 1 Research Institute of Wuhan University in Shenzhen, Shenzhen 518057, China; 2019282020095@whu.edu.cn (M.L.); 2018282020095@whu.edu.cn (C.W.); 2016282020104@whu.edu.cn (P.L.); 2 Hubei Key Laboratory of Ferro and Piezoelectric Materials and Devices, Faculty of Physics and Electronic Science, Hubei University, Wuhan 430062, China; 13554149979@163.com (L.C.); huym@hubu.edu.cn (Y.H.); guhsh@hubu.edu.cn (H.G.); 3 Department of Physics, School of Science, Wuhan University of Technology, Wuhan 430070, China; xiongyaowhut@whut.edu.cn; 4 Key Laboratory of Artificial Micro- and Nano-Structures of Ministry of Education, School of Physics and Technology, Wuhan University, Wuhan 430072, China; gssyhx@whu.edu.cn

**Keywords:** hydrogen, sensing, SnO_2_, Pt, crystal defect, sintering

## Abstract

Many low-dimensional nanostructured metal oxides (MOXs) with impressive room-temperature gas-sensing characteristics have been synthesized, yet transforming them into relatively robust bulk materials has been quite neglected. Pt-decorated SnO_2_ nanoparticles with 0.25–2.5 wt% Pt were prepared, and highly attractive room-temperature hydrogen-sensing characteristics were observed for them all through pressing them into pellets. Some pressed pellets were further sintered over a wide temperature range of 600–1200 °C. Though the room-temperature hydrogen-sensing characteristics were greatly degraded in many samples after sintering, those samples with 0.25 wt% Pt and sintered at 800 °C exhibited impressive room-temperature hydrogen-sensing characteristics comparable to those of their counterparts of as-pressed pellets. The variation of room-temperature hydrogen-sensing characteristics among the samples was explained by the facts that the connectivity between SnO_2_ grains increases with increasing sintering temperature, and Pt promotes oxidation of SnO_2_ at high temperatures. These results clearly demonstrate that some low-dimensional MOX nanocrystals can be successfully transformed into bulk MOXs with improved robustness and comparable room-temperature gas-sensing characteristics.

## 1. Introduction

With such advantages as high sensitivity, high stability, simple operation and low cost gas sensors based on SnO_2_ porous thick films are widely applied for the detection of many reducing gases, including hydrogen and carbon monoxide [1]. However, these SnO_2_ thick film gas sensors all have to work at an elevated temperature [2], which brings numerous problems and is generally regarded as the major drawback for them. In the past few decades, a huge number of research studies have been devoted to developing metal oxides (MOXs) with room-temperature gas-sensing capabilities, among which a large variety of MOX nanocrystals have been synthesized and investigated [3].

While many low-dimensional MOX nanocrystals are very attractive for room-temperature gas sensing, it is a great challenge to characterize their gas-sensing capabilities individually. Usually, they are dispersed in some liquids and dropped on substrates with inter-digital electrodes or mixed with liquids to form pastes and screen-printed on substrates with electrodes. When the liquids are removed through evaporation, MOX nanocrystals are in adequately good contact with one another and with electrodes, and prototypal sensors are thus fabricated to be suitable for gas-sensing measurement. In this way, highly impressive room-temperature gas-sensing capabilities have been successfully observed for many low-dimensional MOX nanocrystals, such as Pd-decorated SnO_2_ nanowires [4], Pd-decorated WO_3_ nanoplates [5], and Pt-decorated SnO_2_ nanoneedles [6]. It has to be admitted that, however, MOX nanocrystals are only stacked together in these sensors, which are of extremely poor mechanical strength and unsuitable for practical applications. Up to date, few investigations have been conducted to improve the mechanical robustness of these prototypal sensors through enhancing the connection between the MOX nanocrystals in them.

Presently, we have prepared a series of Pt-decorated SnO_2_ nanoparticles with Pt in the range of 0.25–2.5 wt%. Through pressing the nanoparticles into pellets, highly impressive room-temperature hydrogen-sensing characteristics have been observed for them all. Such as-pressed pellets are obviously not suitable for practical applications, however, and we have further sintered some of them over a wide temperature range of 600–1200 °C. The mechanical strength is considerably improved through the sintering for all samples, while the room-temperature hydrogen-sensing characteristics are found to vary greatly among them, with strong dependence on both Pt content and sintering temperature. Fortunately, those samples with 0.25 wt% Pt and sintered at 800 °C exhibit highly impressive room-temperature hydrogen-sensing characteristics comparable to those of Pt-decorated SnO_2_ nanoparticles. According to the evolution of the room-temperature hydrogen-sensing characteristics among the samples, it is clearly revealed that the connectivity between SnO_2_ grains increases with increasing sintering temperature, and Pt promotes oxidation of SnO_2_ at high temperatures. These results clearly demonstrate that through composition and sintering optimization, some MOXs in bulk ceramic form not only are able to possess much better mechanical robustness than their low-dimensional nanostructured counterparts, but also exhibit highly impressive room-temperature gas-sensing characteristics comparable to those of the latter. It is meaningful to conduct more investigations to transform more low-dimensional MOX nanocrystals to high-performance and relatively robust room-temperature gas-sensitive bulk MOXs for practical applications in the future.

## 2. Materials and Methods

SnO_2_ nanoparticles (purity 99.99%, 50–70 nm) and H_2_PtCl_6_ 6H_2_O (Pt ≥ 37.5%) from Shanghai Aladdin Biochemical Technology Co., and zinc powder (Zn ≥ 95.0%) from Sinopharm Chemical Reagent Co., were used in this study. In total, 0.1 M H_2_PtCl_6_ solution was prepared from H_2_PtCl_6_ 6H_2_O. SnO_2_ nanoparticles and zinc powder were mixed at a series of designed ratios, and the mixtures were dispersed in deionized water separately through magnetic stirring to form a series of suspensions. For every suspension, 0.1 M H_2_PtCl_6_ solution was slowly dropped to react with the zinc powder in it. After the reaction, the suspensions were centrifuged and dried in oven at 120 °C for 10 h. Pellets of ∼10 mm in diameter and ∼1.2 mm thick were prepared from the dried powders through a hydraulic press. Silver paste was coated on a major surface of some as-pressed pellets to form a pair of rectangular electrodes.

Other pressed pellets were sintered in air for 2 h at 600, 800, 1000, and 1200 °C, separately. Finally, a pair of rectangular Au electrodes was formed on the sintered pellets through DC magnetron sputtering.

Hydrogen-sensing characteristics of samples were measured through a commercial gas-sensing measurement system (GRMS-215, Partulab Com., Wuhan, China) [7]. For the response process, 5% H_2_-N_2_, N_2_, and O_2_ were mixed at some ratios and pumped into the chamber of the measurement system at a total rate of 300 mL/min to simulate desirable hydrogen and oxygen concentrations. For the recovery process, air was pumped into the chamber at a rate of 1000 mL/min from the ambient environment. The room temperature was kept at 25 °C and the relative humidity (RH) in air was kept around 50% for the measurement.

Phase identifications were performed on an X-ray diffractometer (XRD, D8-Advance, Bruker, Rheinstetten, Germany) using Cu Kα radiation. Microstructural observations were conducted through a scanning electron microscopy (SEM, JSM-7100F, JEOL, Tokyo, Japan) and a high-resolution transmission electron microscope (HR-TEM, JEM-2100F, JEOL, Tokyo, Japan). Composition analyses were obtained through energy-dispersive spectroscopy (EDS) using OXFORD Aztec 250 instrument (OXFORD instrument, Oxford, UK).

## 3. Results and Discussions

In the preparation process, Pt nanoparticles were formed through the reaction between H_2_PtCl_6_ and zinc in the following way:

(1) $${\mathrm{Pt}}^{4+}+2\mathrm{Zn}\to \mathrm{Pt}+2{\mathrm{Zn}}^{2+}$$

**Figure 1:**
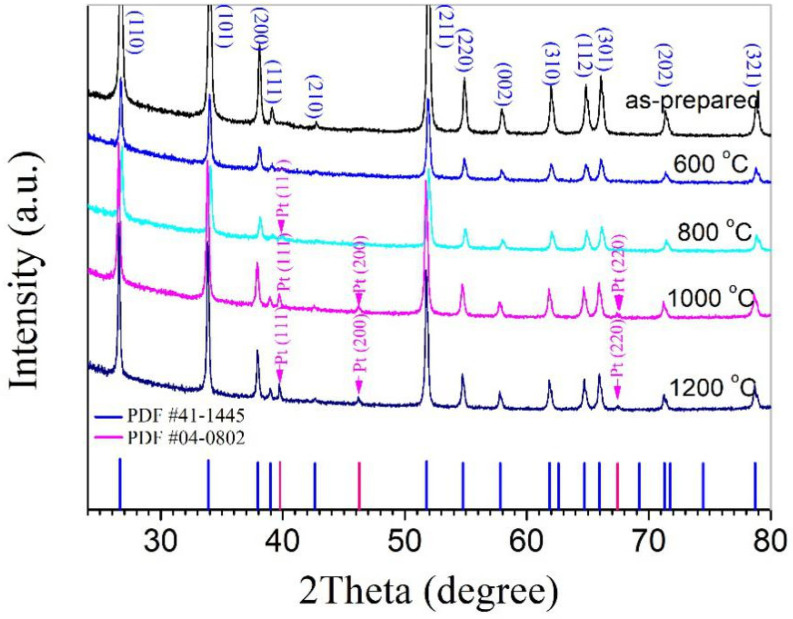
XRD patterns taken for a dried powder mixture of Pt and SnO_2_ nanoparticles with 2.50 wt% Pt as well as pellets prepared from this powder and sintered at 600, 800, 1000, and 1200 °C, separately.

The dried powders, therefore, were mixtures of Pt and SnO_2_ nanoparticles. In this study, a series of mixtures with nominal Pt contents of 0.25, 0.50, 1.00, and 2.50 wt% were designed and prepared. Figure 1 shows the XRD patterns taken for the dried powder with 2.50 wt% Pt as well as pellets prepared from this powder and sintered at 600, 800, 1000, and 1200 °C, separately. For all samples, the strong peaks are identical, and they are from SnO_2_, which exists in a rutile phase according to JCPDS file No. 41–1445. For the two samples sintered at 1000 and 1200 °C, two weak peaks from metallic Pt (JCPDS file No. 04–0802) can be clearly seen, while for the as-prepared powder and the sample sintered at 600 °C, no peaks from Pt can be detected. It is well known that Pt is highly stable and exists in a metallic state after sintering in air at high temperatures such as 1200 °C, so peaks from Pt can be observed in the samples sintered at 1000 and 1200 °C. As for the as-prepared powder and the sample sintered at 600 °C, Pt must be in a relatively low crystalline state, and its peaks cannot be detected for such a low content.

**Figure 2:**
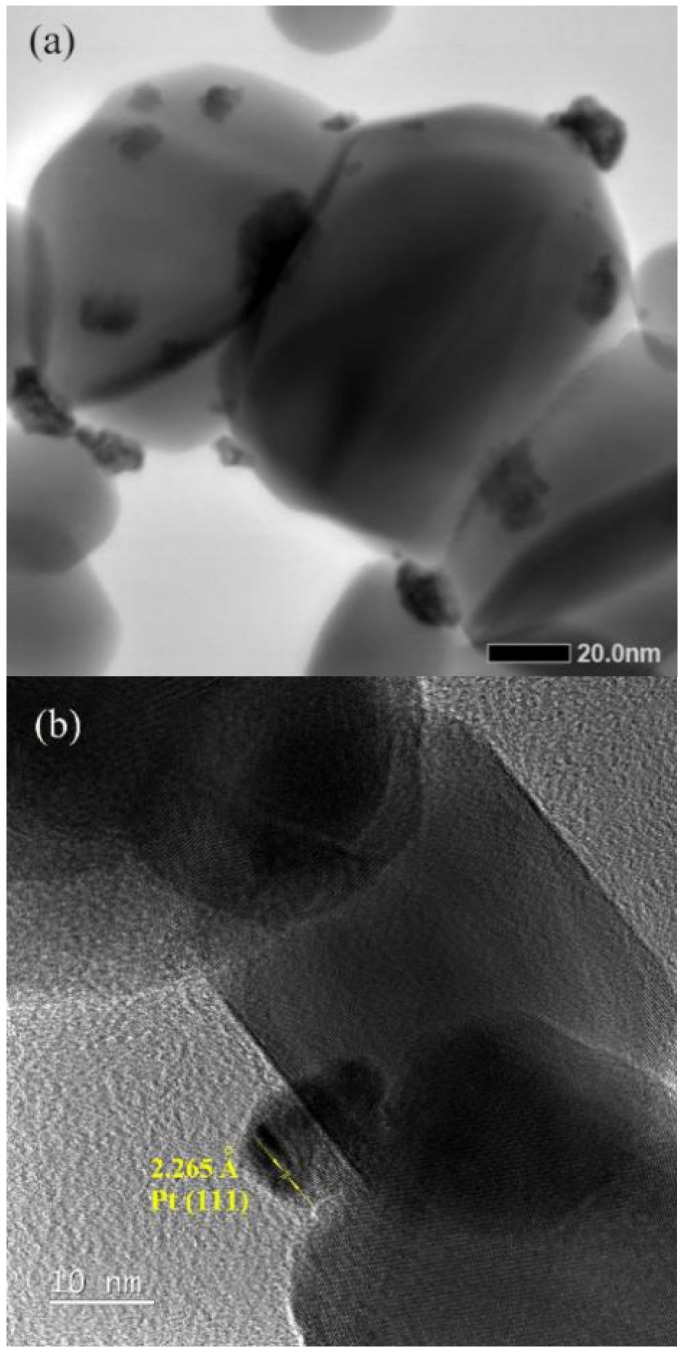
(**a**) A STEM micrograph taken for as-prepared SnO_2_ nanoparticles deposited with 2.50 wt% Pt. (**b**) A HRTEM micrograph taken on a Pt nanoparticle.

Figure 2a shows a scanning transmission electron microscopy (STEM) micrograph taken for as-prepared SnO_2_ nanoparticles deposited with 2.50 wt% Pt. Those nanoparticles of 50–70 nm are SnO_2_ nanoparticles, while those much smaller clusters, around 5 nm in size and very irregular in shape, are Pt nanoparticles deposited on SnO_2_ nanoparticles, as shown by a high-resolution transmission electron microscopy (HRTEM) micrograph in Figure 2b. Both micrographs indicate a low crystalline state of as-deposited Pt nanoparticles.

**Figure 3:**
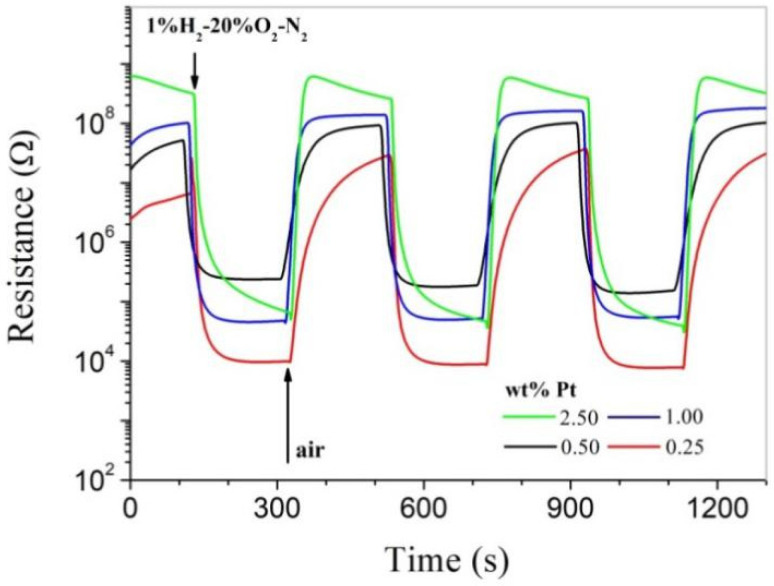
Room-temperature responses to 1.0% H_2_-20% O_2_-N_2_ for as-pressed Pt-SnO_2_ pellets with different Pt contents.

Though Pt was in a low crystalline state in as-prepared powders, highly impressive room-temperature hydrogen-sensing characteristics were observed for all as-pressed pellets, as shown in Figure 3. All samples respond strongly to hydrogen at room temperature, and it can be clearly seen that their resistance in air increases dramatically with increasing Pt content. For the Pd-SnO_2_ system, it has been clearly revealed that Pd is able to promote oxygen chemisorption on SnO_2_ at room temperature [7]. As a similar catalytic metal, Pt should also be able to promote oxygen chemisorption on SnO_2_ at room temperature, which results in increasing resistance with increasing Pt content for the as-pressed pellets. The sensitivity of a gas sensor is usually defined as *R_a_*/*R_g_*, where *R_a_* and *R_g_* are the electrical resistances of the sensor in clean air and in the measuring gas, respectively [7]. The sensitivity is over 100 for all as-pressed pellets, and that of the pellet of 1.00 wt% is around 2000 for 1.0% H_2_-20% O_2_-N_2_, with both fast response and recovery speeds. Obviously, the room-temperature hydrogen-sensing characteristics have been successfully characterized for the Pt-SnO_2_ nanoparticles in this convenient way, which are highly outstanding when compared with those reported for other low-dimensional MOX nanocrystals in the literature [3,4]. On the other hand, however, the as-pressed pellets cannot be regarded as bulk materials and are not stable enough for any practical applications. For example, they will collapse when immersed in water.

**Figure 4:**
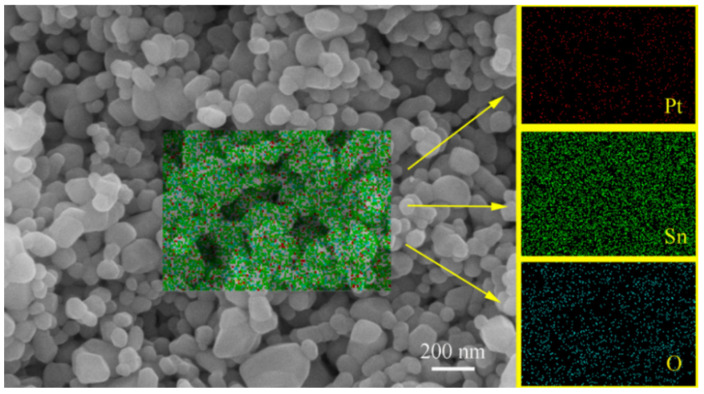
SEM micrograph with EDS analyses for a sample of 1.00 wt% Pt sintered at 1200 °C for 2 h in air.

It is highly desirable to increase the mechanical strength of the as-pressed pellets through sintering while largely maintaining their room-temperature hydrogen-sensing characteristics. For this purpose, we have conducted investigations with a wide sintering temperature range of 600–1200 °C for these samples with a wide Pt content range of 0.25–2.5 wt% [8]. It is interesting to note that there were no detectable shrinkages after sintering for all the samples in this study, including those sintered at 1200 °C. The same result had been observed for Pd-SnO_2_ nanoceramics [7], indicating a quite unique sintering behavior of SnO_2_-based nanoceramics. Figure 4 shows a representative SEM micrograph with EDS analyses obtained for a sample of 1.00 wt% Pt sintered at 1200 °C for 2 h in air. In agreement with the fact of no detectable densification even after sintering at 1200 °C, the microstructure is rich with pores ranging from several dozen to several hundred nanometers. Many grains are around 70 nm in size, and some grains as big as 200 nm can also be seen, which should indicate that grain growth has occurred only in some local areas. Pt nanoparticles prepared in this way are only several nanometers in size [4,7] and cannot be directly observed in the SEM micrograph. According to the EDS analyses, they are rather uniformly dispersed in the nanoceramics.

**Figure 5:**
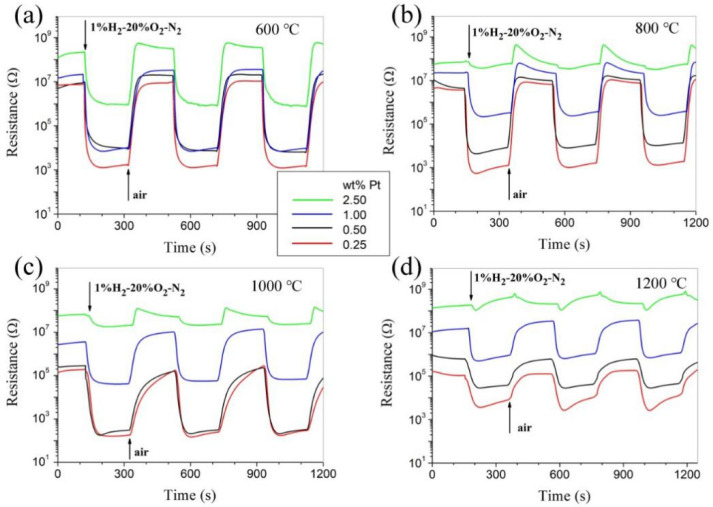
Room-temperature responses to 1.0% H_2_-20% O_2_-N_2_ for Pt-SnO_2_ nanoceramics sintered at a series of temperatures, separately: (**a**) 600 °C. (**b**) 800 °C. (**c**) 1000 °C. (**d**) 1200 °C.

Figure 5 shows the room-temperature responses to 1.0% H_2_–20% O_2_–N2 for Pt-SnO_2_ nanoceramics with various Pt contents and sintered at 600, 800, 1000, and 1200 °C, separately. Though their responses to hydrogen depend greatly on both sintering temperature and Pt content, it is highly encouraging to see that room-temperature hydrogen-sensing characteristics comparable to those of as-pressed pellets are observed for some sintered samples, especially for the one with 0.25 wt% Pt and sintered at 800 °C. With better mechanical strength through sintering, those samples should be much more promising for practical applications than their counterparts of as-pressed pellets. It should be pointed out that SnO_2_-based gas sensors show responses to many reducing gases. These samples with high hydrogen sensitivity in our study were also found to show obvious responses to CO at room temperature. As for the influence of humidity in air, both the resistance in air and the hydrogen sensitivity of the samples decrease with increasing humidity [9].

**Figure 6:**
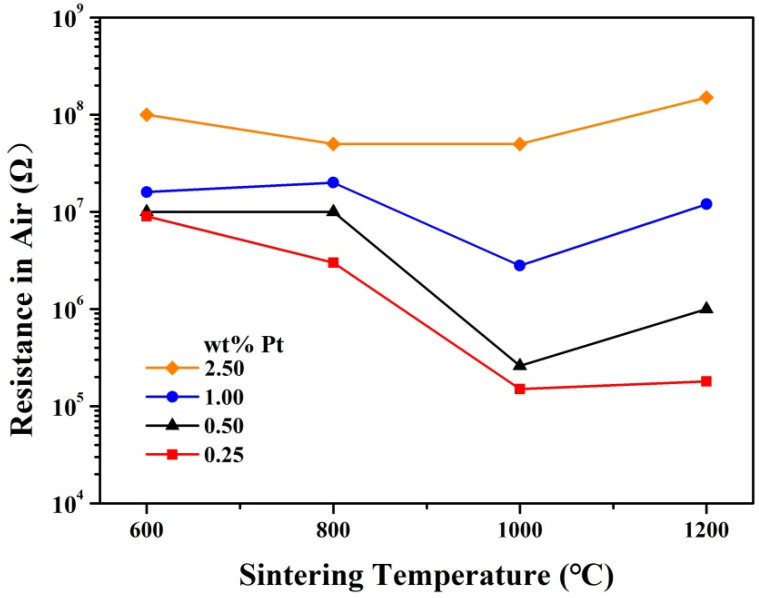
Evolution of resistance in air with sintering temperature for Pt-SnO_2_ nanoceramics of various Pt contents.

The resistance of the samples exhibits a relatively complicated dependence on the sintering temperature. As shown in Figure 6, for samples with 1.00 and 2.50 wt% Pt, the resistance remains roughly unchanged for various sintering temperatures, while for samples with 0.25 and 0.50 wt% Pt, the resistance is much smaller for sintering temperatures of 1000 and 1200 °C than that for sintering temperatures of 600 and 800 °C. Namely, these samples of 0.25 and 0.50 wt% Pt show obviously smaller resistance for relatively high sintering temperatures. To account for this complicated dependence, the resistance of Pt-SnO_2_ nanoceramics should be divided into two parts: The resistance of SnO_2_ grains and that of SnO_2_ grain-boundaries. As the connectivity between SnO_2_ grains increases with increasing sintering temperature, the resistance of SnO_2_ grain-boundaries should decrease with increasing sintering temperature. For samples with 0.25 and 0.50 wt% Pt, the resistance of SnO_2_ grains is relatively small due to the small Pt contents, and their overall resistance must be dominated by that of SnO_2_ grain-boundaries, which decreases with increasing sintering temperature, and the overall resistance is thus much smaller for sintering temperatures of 1000 and 1200 °C. For samples with 1.00 and 2.50 wt% Pt, however, the resistance of SnO_2_ grains is quite high due to the oxygen chemisorption on SnO_2_, which results in the relatively high overall resistance observed even for these samples sintered at 1000 and 1200 °C when the resistance of SnO_2_ grain-boundaries in these samples is greatly decreased. Such an evolution of resistance with sintering temperature should reflect the connecting process between SnO_2_ grains at high temperatures in some way.

**Figure 7:**
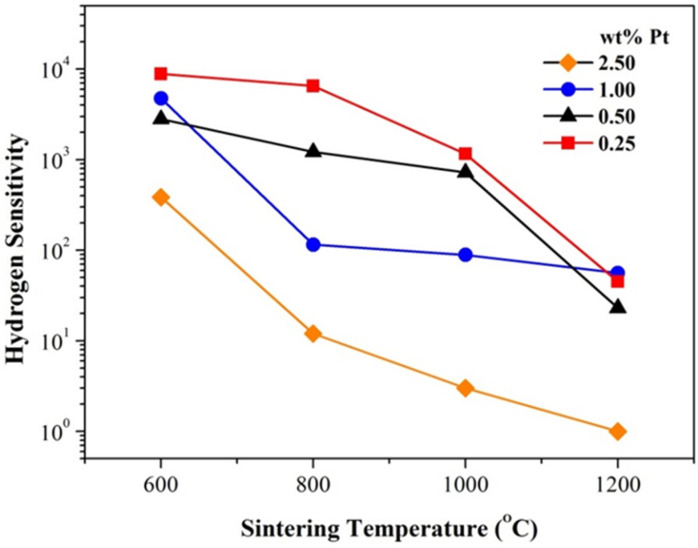
Evolution of hydrogen sensitivity with sintering temperature for Pt-SnO_2_ nanoceramics of various Pt contents.

Sensitivity is one of the most important properties for gas sensors. From the data in Figure 5, it can be drawn that for samples of the same Pt content, the hydrogen sensitivity decreases obviously with increasing sintering temperature, as shown in Figure 7. It should be pointed out that the room-temperature hydrogen sensitivity is still highly impressive for those pellets of 0.25 and 0.50 wt% Pt and sintered at 600 and 800 °C. Obviously, traditional pressing-and-sintering technology has thus demonstrated great potential for preparing high-performance room-temperature gas-sensitive bulk MOXs.

As there were no detectable densifications after sintering for all the samples in this study, such an evolution of hydrogen sensitivity with sintering temperature should not result from densification in sintering, which usually occurs in sintering of most kinds of ceramics. As a matter of fact, such a decrease in hydrogen sensitivity with increasing annealing temperature in air has also been observed for ZnO nanorods, and the decrease in oxygen vacancies was proposed as responsible for it [10]. Oxygen vacancies can also be adopted to account for the hydrogen-sensitivity evolution among samples in this study. The room-temperature hydrogen sensing of Pt-SnO_2_ nanoceramics has been shown to be related to the chemisorption of hydrogen on SnO_2_ at room temperature [11]. It is reasonable to assume that oxygen vacancies in SnO_2_ are able to promote hydrogen chemisorption on SnO_2_. For as-received SnO_2_ nanoparticles, there must be a high concentration of oxygen vacancies in them, and very high hydrogen sensitivity can thus be observed for all as-pressed pellets. For sintering in air, however, the concentration of oxygen vacancies must decrease with increasing sintering temperature due to high-temperature oxidization, which has a negative effect on hydrogen chemisorption and in turn on hydrogen sensitivity. It is worth noting that the hydrogen sensitivity has been most seriously decreased in the sample of 2.50 wt% Pt sintered at 1200 °C. Obviously, such a high Pt content has greatly promoted oxidation of SnO_2_ at 1200 °C. In other words, Pt promotes not only room-temperature oxygen chemisorption on SnO_2_ but also high-temperature oxidation of SnO_2_. When no further annealing in oxidizing/reducing atmospheres is taken into consideration, the sample of 0.25 wt% Pt and sintered at 800 °C should be most attractive for practical applications with both high hydrogen sensitivity and enhanced mechanical strength.

Low-dimensional MOX nanocrystals have been widely investigated for room-temperature gas sensing, in which much attention has usually been focused on their large specific surface areas [12,13,14,15]. As very high room-temperature hydrogen sensitivities have been obtained for all those pellets pressed from Pt-SnO_2_ nanoparticles and for some sintered pellets with optimum sintering temperature and Pt content, our results clearly show that traditional technology of pressing-and-sintering can be adopted to prepare room-temperature gas sensitive bulk MOXs with both high gas-sensing performance and enhanced mechanical strength. Oxygen vacancies in MOXs have important influences on room-temperature gas-sensing properties and can be easily tailored through heat-treatment in oxidizing/reducing atmospheres [16,17,18]. Pressing-and-sintering and special atmosphere annealing should be investigated simultaneously to prepare high-performance room-temperature gas sensitive bulk MOXs in future.

## 4. Conclusions

A series of Pt-decorated SnO_2_ nanoparticles with 0.25–2.5 wt% Pt were synthesized and, after being pressed into pellets, they all exhibited highly attractive room-temperature hydrogen-sensing characteristics. Sintering over a temperature range of 600–1200 °C resulted in obvious degradation in the room-temperature hydrogen-sensing characteristics for many samples, but those samples with 0.25 wt% Pt and sintered at 800 °C exhibited highly impressive room-temperature hydrogen-sensing characteristics quite comparable to those of their counterparts of as-pressed pellets. The effects of sintering on the hydrogen-sensing characteristics of the samples were successfully explained in terms of increasing connectivity between SnO_2_ grains with increasing sintering temperature and Pt promoting oxidation of SnO_2_ at high temperatures. Much attention should be paid to pressing-and-sintering to transform more low-dimensional MOX nanocrystals into bulk MOXs with both relatively high robustness and impressive room-temperature gas-sensing characteristics. 

## Data Availability

Not applicable.
